# Experiences of commissioning services for child and adolescent mental health in England (UK): a qualitative framework analysis

**DOI:** 10.1136/bmjopen-2024-086403

**Published:** 2024-10-29

**Authors:** Kate Allen, Samuel P Trethewey, Frances Mathews, Anna Price, Tamsin Newlove-Delgado

**Affiliations:** 1 University of Exeter Medical School, University of Exeter, Exeter, UK

**Keywords:** Child, Mental Health, Adolescent

## Abstract

**Abstract:**

**Objectives:**

To explore commissioners’ experiences of commissioning services for child and adolescent mental health, their perspectives on the needs of their populations, the challenges they face and their needs for support and data.

**Design:**

Qualitative study involving semi-structured interviews. All interviews were audio-recorded and transcribed verbatim. Data were analysed using framework analysis.

**Setting:**

England, UK.

**Participants:**

12 integrated care board commissioners, responsible for commissioning NHS England Child and Adolescent Mental Health Services (CAMHS).

**Results:**

We identified five themes: *‘reflections on role’; ‘priorities and tensions: working in a complex and evolving integrated care system’; ‘insights and evidence: the role and use of data and informants’; ‘children’s mental health in the limelight: influences and expectations’*; and *‘responding to need "CAMHS as the answer to everything"*’. Combined, these themes highlight the integral role commissioners play in providing oversight over the local system and challenges to this role including disproportionate funding for services for child and adolescent mental health, different use and value ascribed to ‘qualitative’ and ‘quantitative’ data, rises in demand and the limited focus on early intervention and prevention.

**Conclusions:**

CAMHS commissioners are currently negotiating a complex and changing political, social and economic environment with competing priorities and pressures. Our research indicates that commissioners require greater support as their roles continue to evolve.

**STRENGTHS AND LIMITATIONS OF THIS STUDY**

- We conducted interviews with 12 commissioners responsible for commissioning services for child and adolescent mental health (including Child and Adolescent Mental Health Services) from integrated care boards (ICBs) across England; with six NHS regions represented.
- Commissioners participating in our interviews may have been more engaged or research aware than colleagues who did not take part, meaning their perspectives may be different from those not participating.
- As there are only 42 ICBs in England, it is also possible that those participating may not have felt fully able to express their views, due to concerns around anonymity.

## Introduction

 Young people’s mental health has been recognised by the World Health Organisation (WHO) as a global challenge. However, despite the existence of evidence-based interventions for children and young people’s (CYP) mental health, Child and Adolescent Mental Health Services (CAMHS) provision in many countries has fallen far short of need.^1 2^ The process of planning, funding and organising services for CYP with mental health problems varies according to national healthcare funding models and systems. In the English healthcare system, the Department for Health and Social Care sets overall strategy and funding and has oversight of the system, with NHS England acting as the operational arm.^3^ According to NHS England, commissioning is the ‘process of assessing needs, planning and prioritising, purchasing and monitoring health services, to get the best health outcomes’.^4^ In England, the process of commissioning largely sits with regional integrated care boards (ICB), of which there are 42, with primary care and more specialised services commissioned by NHS England.

Commissioners within ICBs have a range of remits, including responsibility for CAMHS. Policies such as Future in Mind^5^ and the NHS Long Term Plan^6^ set out the broad direction of travel for CYP’s mental health, but there remains considerable local flexibility in how services are planned and delivered. Less positively, there has been significant variation nationally in spend and in the level of provision.^7^ Such variation has been found to be only weakly associated with indicators of need.^7^,^9^

Historically in the UK, policymakers and commissioners have been actively hampered in decision-making and service planning for CYP’s mental health by a lack of data regarding national trends and projected changes in prevalence.^10 11^ Since the COVID-19 pandemic, commissioners have also faced challenges in understanding and responding to rapid changes in the context in which services are delivered (eg, the move to online) and the levels of need in their population. These challenges come in addition to pressures to address lengthy waiting lists as well as an increasing policy emphasis on prevention and early intervention.

While some studies have explored how commissioners use evidence in decision-making or policymaking in general,^12^ there is little research on approaches to understanding population need in terms of trends and prevalence. Additionally, to our knowledge, no studies in the English healthcare system have explored commissioner experiences of commissioning CYP’s mental health services post-pandemic. Given the extent and nature of the current challenges in CYP’s mental health, and the central role of commissioners within the system, this appears to be a significant research gap. An improved understanding of commissioners’ needs is likely to have wider benefits for the translation of epidemiological research into practice, by ensuring research outputs meet the needs of key stakeholders and in optimising the sharing, use and interpretation of data to improve services for CYP. Such insights may also be relevant to those researching and delivering services within similar healthcare systems.

This qualitative study aims to better understand English commissioners’ experiences of commissioning CAMHS, their perspectives on the needs of their populations, the challenges they face and their needs for support and data. Our research questions were:

- How do commissioners develop an understanding of the needs of their population?
- How do commissioners plan and adapt services to meet population need?
- What challenges do commissioners face in their roles, and how can they be better supported?

## Method

### Study context

Our study explored the views of commissioners based within ICBs in England who have responsibility for specialist services including NHS England CAMHS, as well as the provision of early intervention support. Commissioners are professionals who will usually have a first degree and postgraduate qualifications, as well as management experience within the health and social care sector. Some may have a clinical background with associated professional registration (eg, as a social worker, nurse or in an allied health profession), but this is generally considered desirable rather than essential.^13^

ICBs are statutory bodies responsible for planning and commissioning healthcare services within NHS England and aim to provide better integrated support across the NHS, local authorities, and community and third-sector organisations to meet the needs of local populations.^14^ ICBs include a chair, chief executive, board members from NHS trusts, local authorities, community and third-sector organisations, and primary care, as well as board members with expertise in mental health.^14^ ICBs are part of broader integrated care systems (ICS), which aim to provide coordinated and collaborative healthcare within 42 regions across England.^14^ ICS replaced clinical commissioning groups (CCG) in 2022 as part of a broader healthcare system reform in England,^15^ which also included the development of ‘provider collaboratives’. Provider collaboratives involve two or more provider organisations (NHS trusts) working together. These collaboratives are intended to ‘blur’ the traditional commissioner/provider split, as they may also take on some of the roles previously associated with commissioners, for example, changing models of care, and signal a more collaborative approach, with less focus on competition.^16^

### Participants and recruitment

We conducted 12 individual, semi-structured interviews with ICB commissioners responsible for commissioning NHS England CAMHS.

We approached and recruited commissioners to take part in the study between May and June 2023 through two main routes: (1) advertisements through social media and commissioner networks; and (2) emails to ICBs within England (UK). Advertisements and emails encouraged interested commissioners to contact the research team. Commissioners who made contact were sent detailed information about the study. Commissioners completed a copy of the consent form prior to interview via email, providing informed consent to participate. Using the concept of information power,^17^ we estimated a sample of 12–16 participants was required to ensure sufficient data to address our research aims and questions.

Of the 12 commissioners who expressed an interest in taking part, all successfully completed a subsequent interview. Details of participants are provided in the Results section.

### Data collection

Individual, semi-structured interviews were conducted online using Microsoft Teams and followed a topic guide designed to address our research questions (see online supplemental material). The topic guide started with questions about the commissioners’ role and their perspectives on the key drivers of CYP’s mental health need, before moving onto questions about how they develop an understanding of needs, how they use data to inform this understanding and the approach they take to planning and adapting CYP’s mental health services. The topic guide ended by reflecting on some of the key challenges commissioners face in their role. All interviews were one-to-one, audio-recorded and transcribed verbatim. Interviews lasted approximately one hour on average.

All interviews were conducted by KA and TN-D between May and June 2023. KA and TN-D are experienced qualitative researchers with experience conducting interviews on mental health and with professionals. Neither KA nor TN-D had any pre-existing relationships with commissioners who took part in the study, however, TN-D does have experience as a public health consultant and has knowledge of the commissioning process and cycle. SPT, who was involved in the analyses, is an academic public health registrar.

### Analysis

We analysed data using framework analysis; a systematic qualitative analysis method which involves charting and organising data into key themes, as well as highlighting patterns within and links between the data.^18^,^20^ The analysis involved several interconnected stages which were conducted by KA and TN-D, with assistance from SPT. KA and TN-D (1) *familiarised* themselves with the interview data by re-reading interview transcripts and any reflexive notes; (2) *coded* the first few interview transcripts line by line using both inductive and deductive codes; (3) *developed an initial analytical framework* based on the research questions, knowledge of theory and prior research, familiarisation stage and line-by-line coding stage; (4) *applied the analytical framework* to the remaining interview transcripts; (5) *charted the data* in a framework matrix; and (6) *interpreted* the data by keeping a regular log of analytical notes and questions throughout the analysis and reflecting on differences/connections between data. KA, TN-D and SPT developed the analytical framework over the course of the analysis and allowed for flexibility to add new inductive codes throughout. NVivo was used to manage the data.

### Patient and public involvement

Both young people and commissioners were involved from the early stages of this research and in the funding application. Both groups contributed to the development of the research questions and the design of the study. For example, young people felt it was important to find out more about how commissioners made decisions about services provided, and commissioners thought it would be helpful to ask about support for commissioners. These were incorporated into the topic guide. From conversations with commissioners, we were also aware of the importance of avoiding the inclusion of identifiable information on participant characteristics as this is a relatively small community of practice. Young people and commissioners were not involved in recruitment to or conduct of the study, or otherwise asked to assess the burden of participation. Commissioners are currently involved in reviewing commissioner and policy-facing outputs. 

## Results

12 commissioners participated in interviews. To avoid identification, details are not provided for individual commissioners. Participants were spread across NHS regions (London n=4; South West n=1; South East n=3; Midlands n=1; North East n=1; North West n=2) and had varying length of experience in a commissioning role (1–7 years, median 5 years). The majority had a remit of child and adolescent mental health and well-being only, but several had a wider remit of children’s health more broadly, or child health and maternity.

**Figure 1:**
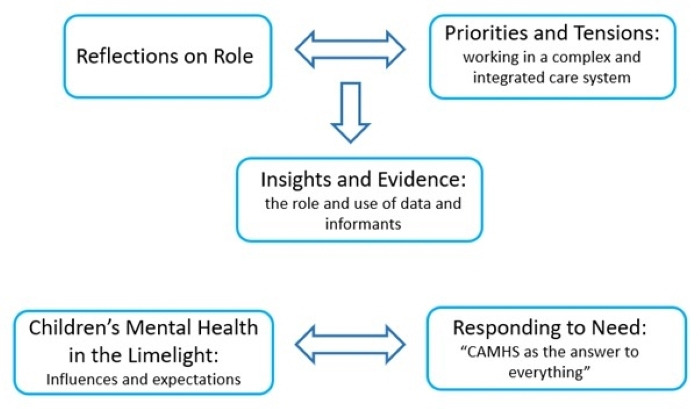
Themes, subthemes and relationships between themes. CAMHS, Child and Adolescent Mental Health Services.

We constructed five key themes (see figure 1): (1) reflections on role; (2) priorities and tensions: working in a complex and evolving integrated care system; (3) insights and evidence: the role and use of data and informants; (4) children’s mental health in the limelight: influences and expectations; and (5) responding to need "CAMHS as the answer to everything".

### Theme 1. Reflections on role

This theme is about the role of the commissioner, how it is informed by their background and their ‘positioning’ in the system in which they work and how this influences their approach to commissioning.

Commissioners had a range of different formal responsibilities in terms of the remit and reach of their roles. They came from a range of different backgrounds, including clinical, non-clinical healthcare, and from professions and organisations outside the NHS. There were different perspectives on how prior experience might inform current role. For example, some commissioners from clinical backgrounds described how they drew on this prior experience in their current roles:

I’m one of the few commissioners who is actually a clinician…So my role as commissioner is actually informed by decades of actual clinical work and also frontline clinical management. (C13)

However, another commissioner described the benefits of having a breadth of prior professional experience:

…(it) allows me to have that kind of broad understanding of a wide range of agendas in making decisions for our communities, which helps. (C10)

Some commissioners clearly ‘positioned’ themselves as ‘non-clinicians’, and linked this directly with how they saw their role or the insights they were able to give:

Because I’m not a clinician, I can’t give you an opinion. I can’t interfere in the way the operational model works. I want to know if there are issues with the way the operational model works. (C8)

However, regardless of background, common to many participants was a perception that one of their key roles was oversight and overview of ‘the system’:

So, because I kind of work at the strategic level, I can see the different parts of the system. Whereas if you work in specialist CAMHS, you just see that bit. You don’t necessarily see, ‘Oh, there’s a school next to me here, and there’s, I don’t know, a school nurse there and there’s the family here.’ You’re kind of just dealing with your one part of it. (C9)

One commissioner described being a ‘*critical point of failure’* (C8) because of this role. In contrast to the commissioning role, providers were often seen as not having the same overview of the system in terms of asking whether they were best placed to offer a particular service. Without their oversight and understanding of connections within the system, confusion or duplication of services could arise, and the system could be fragmented:

And I suppose I see my role, and I don’t think it is really necessarily what my role is per se, but I feel that it should be this, is to try and bring that system together. (C9)

Several commissioners specifically described their roles as supporting system transformations:

I wouldn’t necessarily describe us as traditional commissioners, per se. We work on large scale transformation, so things like the intermediary between NHS England and our organisation, and the subsequent providers that we work with. (C4)

Many described an increasingly collaborative approach to commissioning in recent years, linked to the advent of provider collaboratives (please see Study context for more detail). Commissioners described working together with providers and other organisations to develop consensus around solving problems and addressing gaps. This was closely related to an emphasis on the commissioner’s role in building and managing relationships with providers. Trust, openness and communication were also seen as central to allowing providers to raise problems and difficulties with commissioners:

So, having an open, trusting relationship with providers, where you can just ask questions, or they feel comfortable and confident enough to flag things that they’re finding really difficult, knowing that we will try to work through together to improve a pathway, or to support a team, or whatever it might be. (C7)

Some commissioners contrasted their approach and role with ‘traditional commissioning’ models, which were seen as more adversarial:

So I’m, sort of, part parent, part grandparent, part commissioner, because there is a formal bit to it, I do need to write the specifications, I do need to work with them on their business cases to secure funding…. But I think the fundamental difference is they should feel their commissioners are more approachable, they can talk to them. It’s not transactional, it’s not adversarial. (C8)

However, commissioners also expressed some uncertainties and concerns over their roles. For some, the shift to provider collaboratives meant that they felt their role had changed, or questioned whether they were even supposed to call themselves ‘commissioners’:

…[there needs to be] clarity on the expectations of commissioners, and the commissioning role. Because it seems to be changing, and then assumptions are being made in terms of what the role should be doing. (C6)

So we are not supposed to call ourselves commissioners anymore because the commissioning function has been stripped with regards to the move from CCG to ICS. (C10)

### Theme 2. Priorities and tensions: working in a complex and evolving integrated care system

Commissioners described a wide range of environmental and contextual factors that influenced their work. These factors were perceived to present both opportunities and challenges to the commissioning of mental health services for CYP.

Many commissioners cited key policies and strategies which they felt had influenced their work in their roles. However, commissioners had mixed views about how national policy impacted their work. One commissioner felt a lack of control due to centralised decision-making, whereas another perceived there to be more scope to tailor national policy to local needs:

So we’ll get money from NHS England or money from the central team and they will be like, ‘we’re giving you this money, you have to spend it on X, Y, Z.’ So there’s a lot of stuff that I don’t actually have control over. The stuff that I do have control over is actually really such a small percentage. If anything a lot of the decisions are already made and then just like we’re told we have to just do it. (C7)

I think tailoring national plans, and national objectives, to our local population, is kind of what we do. Obviously we have certain access and activity targets to try and hit, and I guess there are suggestions as to how we do that, but I think what we need to do is really identify how that would work for our local population. (C4)

Participants also discussed the impact of frequent major national healthcare system reorganisations. Several described some of the challenges they faced in adapting to recent system integration and transformation without much information or guidance:

So it keeps on changing from Clinical Commissioning Groups, to ICBs, to Primary Care Trusts, all sorts. I think it’s going to go back to Primary Care Trusts, at this rate. [Laughs] (C6)

Some felt that frequent changes and ‘transformations’, along with a reduction in workforce, had led to a shift towards more reactive commissioning, and to personal stress for commissioners over their own job security:

The demands are unrealistic. Going through a transformation and integration process. We are also going through a restructuring, so we’ve been asked to deliver 30% savings by the end of the next financial year. So my post is safe for this year, but I don’t know if next year there will be a second round of consultation. And this creates a lot of instability because people are losing their jobs, we are going through consultations, so that creates that added stress. (C2)

Related to this, in terms of workload, several participants perceived that they as children’s commissioners had wider remit than they would have in adult’s commissioning:

Resource is not proportionately allocated to children’s commissioning…it’s just not enough. It’s not enough to give the level of attention and the level of time, yes, just the time that is required to do it justice really. (C7)

Some commissioners also felt that CYP as a group were a lower priority in the system. One commissioner directly related this to the change from Clinical Commissioning Groups (CCGs) to ICBs:

(Previously) there was that direct line of responsibility around targeted investment for children and young people and mental health, it felt ringfenced even though it wasn’t but it kind of was because you could not invest in children’s mental health, and commissioning groups were regularly challenged by way of their level of investment through professional bodies, for example, the Royal College of Psychiatrists. For some reason, that connectivity with ICBs, it isn’t the same. I think that’s a major challenge. (C13)

Another felt that other issues were of higher political and media interest than CYP, meaning that young people did not get the ‘airtime’:

Getting airtime to talk about the issues around treatment for young people can be quite difficult because of the operational pressures. You’re on the Six O’clock News with a queue of ambulances outside. Guess what, that’s going to dominate the discussions that week. (C8)

### Theme 3. Insights and evidence: the role and use of data and informants

Commissioners described drawing on a range of sources to develop an understanding of child mental health need and services in their local area. Participants used many different terms to describe these sources, including *information, evidence, data, insight, observations, hunches, conversations, engagement, deep dives,* etc, and had different perceptions on the value and uses of these data.

Service providers were seen as particularly valuable sources of information, being described as commissioners’ ‘*eyes and ears’* (C4). Insights and observations from providers were *key* to ‘verifying’ initial hunches from quantitative data, and prompting deep dives, engagement work or requests for further service provider quantitative data:

I think we are reliant upon our providers, and also our delivery managers, so our borough-based colleagues, to feedback pertinent issues or common trends or presentations, which are either not typical, or they’re increasing. So I think that gives us almost a bit of a hunch. And then I guess what we would do is try to dive a little deeper into some of the activity, outcome and experience data associated with those presentations, or those levels of need. (C4)

This meant that developing strong, trusting relationships with service providers was important, enabling open and honest discussions about problems and levels of need that might not otherwise occur:

[if] they feel comfortable and confident enough to flag things that they’re finding really difficult, knowing that we will try to work through together to improve a pathway, or to support a team, or whatever it might be, is much better than having a dynamic where providers might almost try and consume their own smoke, or to—not hide waiting lists, but feel as though they can’t flag a capacity problem, because they’ll get dragged over the coals for performance issues, and it will just create an industry of perhaps unhelpful dialogue. (C4)

Commissioners also talked of the importance of engagement pieces of work with children, young people and families. This type of information helped commissioners to really understand the needs of their population:

I think the key things that we’ve done in [LOCAL AREA] that I think data will never be able to show you is those engagement pieces of work, so actually understanding what children and young people and families are saying, how they feel about mental health support, what are their needs. (C12)

However, this was not always the case, with one commissioner suggesting that engagement events or co-production was not always helpful as it did not tell them anything new:

[Talking about feedback from children and young people co-production and engagement events] I wouldn’t say that they are to give us a good granular detail direction for developing services, because the narrative is always the same, ‘Oh, I wait too long, I don’t want to go to CAMHS, I want to be seen in the community,’ these are the main themes. (C1)

Commissioners also reported using ‘hard’ quantitative data (eg, service provider data, population-level data, local surveys, academic data) to develop an understanding of child mental health need and identify gaps in service support. Commissioners talked about population data being useful in providing a spotlight on general need, evidence for business cases (with quantitative data carrying more weight than qualitative data) and looking beyond ‘levels of access’ data:

I think looking at prevalence rates in boroughs and PCNs, versus the actual activity or access levels, is able to shine a bit of a light on where we have high level of need, but perhaps low levels of access. (C4)

I wouldn’t need it day-to-day, no, but we would obviously access that when we’re looking at papers or bidding or business cases, recommendations or retendering, all of that type of stuff then yes, or writing papers and we need that information, like going on the JSNA and things like that. (C3)

Often, there was a reliance on service provider data (ie, data on referrals, activity, outcomes and wait times), over other forms of quantitative data, to inform commissioners’ understanding of child mental health need. Service provider data had a range of uses; from identifying emerging context-specific trends to assessing service performance and identifying where services might be failing to meet the needs of CYP. However, some commissioners had concerns about the reliability and accuracy of these quantitative data. For example, service provider data were often reported as missing, coded inaccurately or inconsistently captured. This led to a sense that these service provider data could not always be trusted to inform decision-making, and qualitative data were needed to *truly* understand child mental health need and to ‘*improve [commissioners] view of the accuracy of a numeric’* (C8):

[quantitative] data informs us maybe of some of the work that we need to do, and the areas in which we need to do that work. Then from there, and again, we wouldn’t do this in isolation, as an ICB, we do this with our colleagues, from services, to understand, okay, what is the need? And it may be that we need to do an engagement piece of work in that area. (C5)

The pressure on service providers to provide quantitative data was seen by one commissioner as having unintended consequences; encouraging service providers to chase referral numbers rather than provide quality support. Furthermore, concerns around the reliability, accuracy and novelty of quantitative data led some commissioners to ascribe little value to this type of data:

It’s an odd one, [name] because if you talk to people in health, they’ll say, ‘Everyone knows where the problems are. So why do we need more data to tell us? It’s got feathers like a duck and it quacks like a duck. It’s a duck. We know where we need to make the changes. We don’t need more data or more surveys or more.’ […] ‘I kind of know where the problem is, and I don’t have to have empirical data to help me with that.’ (C8)

There was a clear tension and variation between the use and value of quantitative versus qualitative data. Commissioners’ background and experience often influenced the type of data they used (eg, commissioners with a quantitative background talked about using and valuing quantitative data, whereas commissioners who were clinicians by background often ascribed greater value to initial hunches or conversations with service providers). As a result, this created a commissioning landscape in which there appeared to be no established way of using data and informants; with sources/type used varying, dependent on the individual commissioner.

### Theme 4. Children’s mental health in the limelight: influences and expectations

This theme is about commissioners’ perceptions of changes in child mental health in their populations. Most commissioners discussed having seen a rise in demand for services, in terms of referrals and increasing time on waiting lists. Participants listed a range of areas where they felt there had been increases, including neurodevelopmental disorders, disordered eating, depression and anxiety, and self-harming. Many also perceived an increase in complexity and urgency of presentations. Commissioners made a clear link with the impact of the pandemic, but also emphasised that rises in demand were already happening pre-COVID:

I don’t want to pin it all on COVID because we were seeing an increase in demand before COVID. So it’s not as if everything was fine and then COVID, you know, just tipped us over. (C9)

Participants felt that social isolation and online education were key factors that had affected mental health and child development and contributed to school-based anxiety and avoidance. However, commissioners also voiced concern over the impact of other systemic social and economic influences which they felt were driving increases in problems, and which had been exacerbated by the pandemic:

I might be wrong, but I don’t think that there has been a biological shift in how children and young people’s mental health and emotional wellbeing works. I think that a lot of it’s from external factors. (C4)

Inequalities in access to help were also a concern:

I think the pandemic created a larger gap between those who needed help, or need help, and those who access it. I think that’s been disproportionate for certain children and young people, certain demographic profiles as well. (C4)

There was also much discussion of the effects of increased awareness and understanding around mental health, and the impact of social media and wider policy in putting mental health ‘*in the limelight’*. This was also seen as uncovering new needs and gaps which services then had to meet:

I would say that the demand has increased, that’s a fact because we’ve expanded services and still have issues with waiting times and more children needing support. But alongside the demand, because mental health has increasingly been in the limelight, I suppose, there’s been more attention to mental health, child, adolescent, mental health in the last five years. Perhaps this has uncovered needs and a prevalence that we weren’t aware of before. (C1)

Disordered eating and things like ARFID [Avoidant Restrictive Food Intake Disorder], seem to be something… sort of newly emerging conditions because we don’t have commissioning in place for them. And that’s not because it was a gap… well, it’s a gap now, but it’s a gap that seems to have emerged within recent times. When we, for example, were commissioning our specialist children’s mental health service, disordered eating and ARFID were not really discussed as being something that needed to be considered or perhaps disordered eating was looked at differently. (C2)

In terms of drivers of demand, commissioners also appeared to perceive some tensions between positive aspects of awareness and encouraging help seeking, and risks of ‘over-medicalising’ normal ‘ups and downs’ and expectations of mental health which might be unrealistic:

So there’s an awareness from children and young people that they know what good mental health looks like, and how they can promote it. But there’s also probably an expectation from that as well…I think a young person, after watching a video, or trying to learn some coping techniques, won’t necessarily see a change or an improvement straight away in the same way that, if you’ve got a headache, you have a paracetamol, and then an hour later you’re sorted… (C4)

### Theme 5. Responding to need: "CAMHS as the answer to everything"

In addressing the needs of their populations, many commissioners felt that prevention and early intervention were key priorities within child and adolescent mental health. They discussed an increasing role for a broader range of agencies including the voluntary sector, social care and education, with several referencing the THRIVE model (a needs-led framework to help create communities of mental health and well-being support, with a focus on proactive prevention and promotion).^21^ Participants also cited a range of initiatives aimed at triaging referrals and expanding first-line support and emphasising the role of CAMHS as a specialist mental health service. However, for some commissioners, there were several significant challenges in moving the focus away from specialist child mental health services. For example, lack of capacity in other parts of the system such as schools and communities was seen as a limiting factor in prevention and management of less severe cases, resulting in bottlenecks and pressure on CAMHS:

I think there is a real-time reduction in staff time in schools over the past 15 years and that gets expressed in an increased in referral activity to where the lights are on. That’s the NHS. And free at the point of use. So there is a systemwide bottleneck and things just breaking down. (C13)

Additionally, commissioners perceived that both professionals and young people still had assumptions and expectations that CAMHS was the place for all child mental health concerns. These expectations complicated efforts to develop other pathways, with some early-support services being seen as underused as a result of a preference for CAMHS:

We get reports from services that they are under strain, but there are also parts of other services which are not utilised properly. And then we get feedback from children, ‘I don’t know what provision is available to me, and waiting times are too large.’ So my feeling is we’re still struggling to move the focus away from CAMHS being the answer to everything. (C1)

Some expressed frustration that this might mean CYP spent time on waiting lists when they could have accessed support more quickly elsewhere:

There’s still that mind-set of if someone expresses any concern about mental health, let’s refer them into CAMHS, and that’s where we see those waits because then they’re essentially on a waiting list for support that potentially they might not need and they could have got something a lot earlier. (C6)

Commissioners also reflected on future directions and challenges and what they expected to see coming down the line. One commissioner thought that funding would never be able to meet need for services:

The overarching difficulty is that the money that we’ve got is never going to be able to meet the full demand of need, so we have to think differently about who we support when. (C12)

Commissioners had varying degrees of optimism about whether investment in services and early intervention would happen, and whether CYP’s mental health would improve in future:

Our focus needs to be more on that preventative and early intervention. But that’s a massive culture change, so I think hopefully in that long period of time we will see those—I won’t say probably a reduction—but levelling off of demand. (C12)

I would say unless, coming back to the main point, we invest properly and proportionately in children’s services over the next five years, we’re going to be going backwards in terms of children and adolescent mental health. We’re not going to be going forwards. […] So it sounds pessimistic, but I think we’re going to have a worse situation in the next five years unless we drastically change. (C7)

## Discussion

This qualitative study sought to explore and understand commissioners’ experiences of commissioning child mental health services, their perspectives on the needs of their populations, the challenges they face and their needs for support and data. From interviews with 12 commissioners based in ICBs across England, we generated five themes: (1) reflections on role; (2) priorities and tensions: working in a complex and evolving integrated care system; (3) insights and evidence: the role and use of data and informants; (4) children’s mental health in the limelight: influences and expectations; and (5) responding to need: "CAMHS as the answer to everything". Below we discuss some of the main insights, challenges and implications of these findings.

Our first and second themes illustrate the way in which commissioners see themselves as holding oversight or a living map of their local systems, and have an increasing role in systems leadership, and in collaborating with providers, rather than focusing on a more adversarial system of contracts management. Many seemed to feel this enabled greater openness over problems and shared challenges. However, it was also evident that some commissioners experienced tensions and uncertainties over their changing roles, especially when these changes occurred as part of broader restructuring. While some benefits were seen, there was a strong perception that frequent reinvention and transformational change had introduced stresses about job security, and hampered their being able to undertake deeper thinking and proactive planning over the longer term. Here our findings are in line with previous research suggesting that whole system changes can result in disruptions to the commissioning process, as well as to the wider workforce.^22^ Such healthcare services reorganisations are not unique to the English context, with one study reporting on 78 reforms being implemented across 26 European countries, clustering around to changes to ‘coverage & resource generation’, ‘purchasing & payment’ and ‘hospital care’.^23^ While there is often interest in the impact of reorganisation on clinical staff and patients, our findings suggest that attention should also be paid to the impact on those responsible for planning and managing services in a commissioning role.

Commissioners also faced challenges in terms of advocating for their population of CYP. Many of our participants perceived that CYP as a group were seen as lower priority, both in terms of allocation of commissioning resource, but also in the healthcare system. This accords with wider concerns which have been repeatedly voiced about the (relative) low priority of CYP in policy and strategy in England, and even in the COVID-19 response.^24 25^ Related to this, there were mixed reports from commissioners about the extent of their agency within the system, and how much they could tailor national policy to local need, which suggests that system-level factors within different ICS may affect the scope of commissioners’ decision-making.

A key finding from our third theme, ‘Insights and evidence’, was the various ways in which commissioners developed an understanding of the needs of their populations, and how they perceived the roles of ‘qualitative’ and ‘quantitative’ data. Many participants placed a premium on the insights they gained from trusted providers acting as their ‘eyes and ears’. This constituted another benefit for commissioners of developing good relationships with their provider networks. Evidence gathered from engagement with children and families through local groups and events was also seen as providing strong narratives and stories which could spotlight areas of need, although the degree to which this process involved underserved groups in the community, as opposed to highly engaged ones, was unclear. Other research on policymaking and commissioning has also highlighted the importance of ‘people based’ sources of information, often as part of informal ‘policy networks’.^12 26^ Interestingly our research also reveals the concerns held by commissioners about ‘quantitative data’. In some cases, concerns around the reliability, accuracy and novelty of quantitative data led some commissioners to see quantitative data as being lower value. Despite this, most saw a clear role for quantitative data, and would expect to include it in documents required for decision-making such as in needs assessments and business cases.

Commissioners’ perceptions of changes in their populations were in many ways in line with findings from epidemiological data, in terms of the gradual increase in prevalence pre-COVID, followed by a more marked rise in problems, particularly in emotional disorders.^27 28^ However, commissioners also discussed how and whether increased awareness and understanding of mental health were influencing presentations and demand for services in their areas. ARFID was cited by at least one commissioner as an example of a ‘new need’ that had been uncovered and which services needed to meet. These discussions appeared to reflect wider international societal debate on the impact of broader mental health awareness and the medicalisation of distress in young people.^29^

Linked to this was the theme on ‘CAMHS as the answer to everything’, which described how commissioners were grappling with system transformation (mirrored by other accounts from CAMHS staff, eg, in Fazel *et al*’s^30^ study). A common area of challenge appeared to be the challenges of diverting the focus from CAMHS for problems which may not require specialist support. Some participants perceived that early-help or online groups were underused due to a preference for CAMHS both from referring professionals and from young people and families. This presents an interesting contrast with research suggesting that patients and primary care professionals perceive marked barriers to accessing care and that thresholds for CAMHS remain a hindrance in getting the support they need.^31^,^33^ The accounts of commissioners accord with the wider drive within ICS to change the focus of services to include lower intensity early interventions to support health and well-being in the population.

### Strengths and limitations

One of the strengths of this paper is the exploration of the perspectives of commissioners, a group who are rarely the ‘subjects’ of research, but who have considerable influence in the design and delivery of mental health services for CYP. This study represents one of only a handful of papers to explore their views, and the only study we are aware of to include CAMHS commissioners in a post-pandemic context. We were also able to recruit commissioners across a range of ICBs in England with responsibilities for commissioning mental health services for CYP. The limitations of the study include the potential for more motivated and research-engaged commissioners to have participated, perhaps affecting the transferability of our findings. It is also possible that those participating may not have felt fully able to express their views, due to concerns around anonymity. As there are only 42 ICBs in England, we have been careful to avoid including information which could link a participant to their ICB and risk disclosure.

### Implications

Our findings describe the complex and changing political, social and economic environment in which commissioners work, and their role in maintaining oversight of their local systems. It is also evident that commissioners as a group may struggle with changes as their role evolves, and that they may benefit from additional resource, training and professional development opportunities. As with many public sector services, CAMHS commissioners also face challenges in delivering services in the face of constrained resources. Marked tensions were evident around the perceived need to balance investment in prevention and interventions earlier in ‘the pipeline’ with continuing to deliver the specialist assessment and treatment needed by children with more severe and complex problems. While there have been long-standing calls for more investment in child mental health since before the pandemic, it has been argued that services are now even more unlikely to be able to meet growing need without a step change in thinking by those funding and designing services.^34^ Decision-making in this context needs to be as transparent as possible to all stakeholders (including the public), including developing a clearer understanding of the data required and used to make difficult commissioning decisions. Similarly, our findings on the variation in approaches to commissioning raise questions about the need for more standardised procedures and guidelines, while recognising the importance of local insights.

These findings also have important implications for researchers, in terms of how those in the research community can ensure that relevant and digestible messages find their way to those commissioning services. Our findings highlight how time, and timeliness, is of the essence for those in a commissioning role, hence brevity and pace of outputs is key, representing a challenge for researchers working with slower timescales of funding, approvals and peer review. Local relationships and networks are likely to be key for research teams to interact in a more meaningful way with commissioners. Forums that bring the two groups together, for example, as part of a research active ICS or population health management board, may also be helpful in exploring joint priorities and co-development of outputs. Finally, while these specific findings are situated in the English healthcare system, we would suggest that many other healthcare systems internationally are facing similar challenges regarding how to plan and deliver services for CYP’s mental health, and hence, some of our findings may be translatable and transferable in an international context.

## Conclusions

Our study sought to explore and understand commissioners’ experiences of commissioning services for child and adolescent mental health, including their perspectives on the needs of their populations, challenges they face and their needs for support and data. The findings highlight how commissioners are negotiating a complex and changing political, social and economic environment with competing priorities and pressures. Proportionate funding for CYP’s mental health services was seen by commissioners as essential to ensure services are able to meet current need, alongside a greater focus on prevention. Researchers now need to work alongside commissioners to provide timely, succinct outputs that better support commissioners’ plan services and improve the health of the populations they serve.

## supplementary material

10.1136/bmjopen-2024-086403 online supplemental file 1

## Data Availability

No data are available.
