# Supplementary material for: Experiences of commissioning services for child and adolescent mental health in England (UK): a qualitative framework analysis
Source: BMJ Open. 2024 Oct 29;14(10):e086403. doi: 10.1136/bmjopen-2024-086403 (PMC11529587; doi:10.1136/bmjopen-2024-086403)
Supplement: online supplemental file 1 [file bmjopen-14-10-s001.pdf]

Supplementary material

Interview topic guide

1) Can you tell me a little bit about your role as a commissioner?
    a. Which services are you currently responsible for commissioning?
    b. How long have you been in your role as a commissioner?

2) What recent changes have you seen in child mental health need? (nationally/locally)
    a. In your view, what are the key drivers/determinants/factors responsible for these changes?
    b. How do you think COVID played a role in these changes?
    c. Can you tell me about any specific groups that might have been impacted?

3) What changes do you expect to see in child mental health need moving forward? (e.g., next five/ten years?)
    a. In your view, what are the future drivers/determinants of child mental health likely to include?
    b. How do you think CAMHS might need to adapt to meet these changing needs?

4) How do you develop an understanding of child mental health needs in your area?
    a. Can you talk me through the process involved?
    b. Can you tell me about who you work with to help develop this understanding?

5) How do you use data to help inform your understanding of local child mental health needs?
    a. What sources of data do you use?
    b. Can you describe what these sources of data include?

c. How do you find accessing/using this data?

d. What is *useful* about this data? What is *not so useful* about this data?

e. Are there any sources of data that you do not use? Can you tell me about why you do not use this data?

6) What approach do you take to planning and adapting child mental health services in your area?

   a. What resources do you draw on?

      i. How does this help inform your decision-making?

   b. What expertise do you draw on?

      i. How does this help inform your decision-making?

   c. Can you tell me about how you work with others to plan and adapt child mental health services?

   d. What differences are there, if any, in your approach to planning and adapting mental health services for transition age young people?

7) How could the data currently available to you be improved to better support you in your role?

   a. What are the key gaps with the data?

   b. What are the key problems/limitations with the data?

   c. What additional information would you like to see in the data currently available? Can you tell me about why this data would be particularly important/useful?

   d. Do you have any key unanswered questions about your population?

8) Can you tell me about any specific groups that it would be useful to have data for?

9) What are the key challenges you face in undertaking your role as a commissioner of child mental health services?

10) Is there anything else that would be useful to you in terms of commissioning child mental health services?
