# Supplementary material for: Experiences of commissioning services for child and adolescent mental health in England (UK): a qualitative framework analysis
Source: BMJ Open. 2024 Oct 29;14(10):e086403. doi: 10.1136/bmjopen-2024-086403 (PMC11529587; doi:10.1136/bmjopen-2024-086403)
Supplement: Supplementary file 2 [file bmjopen-2024-086403.reviewer_comments.pdf]

PEER REVIEW HISTORY

BMJ Open publishes all reviews undertaken for accepted manuscripts. Reviewers are asked to complete a checklist review form and are provided with free text boxes to elaborate on their assessment. These free text comments are reproduced below.

ARTICLE DETAILS

Title (Provisional)

Experiences of commissioning services for child and adolescent mental health in England (UK): a qualitative framework analysis

Authors

Allen, Kate; Trethewey, Samuel P; Mathews, Frances; Price, Anna; Newlove-Delgado, Tamsin

---

VERSION 1 - REVIEW

**Reviewer**	**1**
**Name**	**Cui, Chengsen**
**Affiliation**	**Peking University, China Center for Health Development Studies**
**Date**	**26-Mar-2024**
**COI**	**I confirm that there is no competing interest.**

1.Major comments

(1) About the contribution

What are the policy implications of CAMHS for other countries? Why should international readers care about CAMHS, and what is its role in the health system? Although the study highlights the need to provide timely, succinct research outputs designed to support commissioners and proportionate funding for children and young people's (CYP's) mental health services and a greater focus on early intervention and prevention, it is only based on the UK context.

(2) Introduction

i. The authors need to explain more about why Mental Health Services are essential rather than the fact that there is such a CAMHS.

ii. The background section should not just review CAMHS studies but should include relevant papers on similar mental health services in other countries to support the contribution of this study.

(3) Method

i. The author does not explain how the five key themes are constructed. The author must describe the building steps or the conceptual framework referenced in detail. Otherwise, these five points are arbitrary and illogical.

(4) Results

i. I suggest presenting the information of these 12 interviewees in the table and disclosing more critical information that can reflect their representativeness, such as professional background, which is very important for this study.

ii. The author should use Nvivo to analyze and present some primary quantitative data, such as crucial word frequency, which can make this study more complete. In the current manuscript, Nvivo is not necessary.

(5) Discussion

The authors confine themselves to discussing the CAMHS system without whether it can be used in other countries. As mentioned in the title of this article, this is a study based on experience. However, the current manuscript has not yet achieved the author's purpose.

2.Minor comments

The references lack evidence from high-quality journals, significantly research articles.

**Reviewer**	**2**
**Name**	**Hellner, Clara**
**Affiliation**	**Karolinska Institute, Clinical neuroscience**
**Date**	**19-May-2024**
**COI**	**None**

This is an interesting paper but it lacks enough overview and analytical perspective in order to make it interesting for an international audience.

Comments on ms bmjopen-2024-086403
”Experiences of commissioning services for child and adolescent mental health in England (UK): a qualitative framework analysis

Thank you for allowing me to review this interesting paper, that touches upon the important issue of organizing mental health services for children and young people. The study focuses on an important part of the provision chain, i.e. those professionals that serve on the commissioning side.

General comments
My main comment has to do with the lack of “packaging” the study in a way that makes it understandable for an international audience. The framework of health systems research

seems a bit absent. As a non-UK mental health professional, I ended up having many questions after reading, for example:

How is the provision of care organized in the UK? I know of the NHS, but how does it work in practice? Who decides, and on what level, what is to be prioritized and what resources are allocated to the regional/local level? Policies are referred to, but it is unclear if these are just recommendations or if it they are expected to be followed. How is the concept of evidence base incorporated in policies? Are there political decisions somewhere along the way?

How is the responsibility for CYP mental health divided between the primary care level (GP) and the specialist level? Do schools have any responsibility for child health/child mental health? Are these commissioners involved only on the specialist care level, and if so – how do they communicate with other parts of the service provider system and decide who does what?

Please describe how an ICB is organized and what type of professionals usually are part of such boards and how they work. Even though it is justifiable not to describe the specifics of each informant in the paper, more contextual knowledge of the commissioner professional profile, role and mandate would help. It appears from lines 184-188 as if the commissioner role can be filled by various types of professionals – but who decides what competence is required? Any national guidelines/recommendations? Given that variability across the country is described this made me curious.

A detail: I am not a native English speaker and had some difficulty to understand the term “remit” that is widely used. My dictionary did not help much but I interpret the use of the word as if it intends to mean mandate or range of responsibility– correct?

What type of background are these professionals expected to have? What is the relationship between the individual commissioner and the care provider? How much responsibility has an individual commissioner and how much are they expected to just deliver “on order”? You describe a shift in roles towards more dialogue, but is the key responsibility to produce contracts? Without such a contextual understanding, it is hard to interpret the results.

Objective and Method
An important part of qualitative research is to clarify the perspectives from which the study has been performed, i.e. the motivations for doing the study. Throughout reading the paper, I sometimes wonder if the purpose is to highlight the situation and complexity of a certain professional role (“professional wellbeing”), or to understand the process of commissioning services for CYP mental health is (“systems approach”). Both perspectives are justified but motives need to be clarified. It is stated that the objective is “to explore commissioners’ experiences…” but still that can be done in relation to both aims outlined above.

With a self-selection procedure the limitation always is that the group of informants may not be representative, but that has been highlighted. Were all the informants selected and decided at the same time point, or did the authors consider adding more informants until saturation occurred? Did the authors consider triangulating the data collection and also do a survey, or to interview professionals with leadership responsibility in the provider chain?

Line 155-57: I did not understand sentence “Commissioners have provided input into the key messages developed from this study and are currently involved in reviewing commissioner and policy-facing outputs”. Please clarify whether “commissioners” (informants or other

commissioners) have influenced the interpretation of results of the study in any way (i.e. in this manuscript).

The study was financed by various grants, which are described by abbreviations (e.g. line 652 “TND’s NIHR Advanced Fellowship” and “NIHR Academic Fellowship”). Please clarify what these refer to more clearly and also if the funding institute in some way had some interest in the outcome of the study, and/or if they were involved in the design, performance, or interpretation of results in any way.

Results
The results are described pretty straight forward. Themes were understandable and the quotes in most cases related to the themes. A few comments:

Line 232: “…linked to the advent of provider collaboratives”. It appears as if authors here refer to a common trend or was it just this commissioner who described a change in how s/he worked? Further down on the same page the term “traditional commissioning models” is mentioned. Should I understand this as if the traditional model was a pure procurement procedure and that this traditional model is being abandoned on a larger scale?
(this is a good example when lack of insight into the UK model makes it harder to interpret the text)

Line 283: The term “frequent major healthcare system reorganisations” is mentioned. Does the informant here refer to local reorganisations or is this something that changed all over the country at the same time?

Line 477-478: “…many commissioners felt that prevention and early intervention were key priorities”. Do they refer to their own activities here or do they point in other directions/at other organisations? This is also mentioned in a quote on line 520.

Line 479: Since the authors state that several informants referred to something called the Thrive model, it could facilitate for the reader is this model was briefly described here as well (and not only referred to)

Discussion
The discussion still is quite descriptive and repeats/summarises results more than analyses them and puts them into context.

One merit of this paper is that it focuses an important part of the complex process of providing mental health services to CYP. However, with the lack of a clearer description of the analytical framework guiding the paper, it appears as if the interpretation of the findings mixes the systems with the professional role perspective.

The second and third paragraph (540-555) discusses the various perspectives on the commissioner role – system oversight/leadership or contract management? Once this dual role is identified, as it is in the results part, it would be interesting to *discuss* the duality rather than just describing it again. For example: Is it even possible to exercise system leadership while at the same time “buying” services from it? If so, how should commissioners interact with the system and still protect system integrity? Are there any risks identified in relation to this duality? And how are patients’ interests protected in the process?

Lines 557-580 discusses the process of identifying needs and commissioning services according to such needs. The interesting part here is the described variation in how the informants work, what data they have access to, how they value/not value certain types of data etc. Here a more system oriented discussion could be warranted. The observation (i.e. description of such existing variation) justifies a discussion if there is need for system development. Based on these findings, is there a need for a broader discussion on a standardized procedure/guidelines for commissioning services? I do not see the stakeholder perspective here, the focus is more on the challenges related to the professional role of being a commissioner. But what can stakeholders (i.e. children, youth and their families) expect in terms of how decision makers and commissioners arrange the way they work? Would it be relevant to recommend triangulation of data (epidemiological data, provider data, and dialogue with stakeholders as well as experts) before deciding on such important issues as service provision for mental health in youth?

Lines 582-592: This paragraph also describes part of the health systems pointing at each other. This is a well known phenomena also in my country. Here as well, it would be possible to discuss how the “system” could interact/develop in ways which ultimately would see to the whole care chain and how each part can contribute to whole. Are there any examples of alternative ways of working or organizing services that would make it easier for CYP to access services according to needs? I do not expect ultimate solutions anywhere, but maybe good (or better) examples can be identified? Or attempts to cross bridges between piecemeal solutions?

VERSION 1 - AUTHOR RESPONSE

<u>Response to reviewers</u>

Comments and suggested changes	Author response
**Reviewer 1**	
**<u>(1) About the contribution</u>**<br>What are the policy implications of CAMHS for other countries? Why should international readers care about CAMHS, and what is its role in the health system? Although the study highlights the need to provide timely, succinct research outputs designed to support commissioners and proportionate funding for children and young people’s (CYP’s) mental health services and a greater focus on early intervention and prevention, it is only based on the UK context.	Thank you, we have provided additional context on the importance of child mental health globally and the ‘service gap’ in the Introduction. Whilst our study is situated in the UK context, in the Discussion we draw out implications for international audiences.
**<u>(2) Introduction</u>**<br>i. The authors need to explain more about why Mental Health Services are essential rather than the fact that there is such a CAMHS.	Thank you. Please see comment above regarding the addition of further context.
ii. The background section should not just review CAMHS studies but should include	Thank you. Given the study is in a UK context, we have not added extensive literature on the state of

relevant papers on similar mental health services in other countries to support the contribution of this study.	CAMHS in other countries, but cite a relevant review of the state of CAMHS globally in our expanded introduction.
**(3) Method**<br>i. The author does not explain how the five key themes are constructed. The author must describe the building steps or the conceptual framework referenced in detail. Otherwise, these five points are arbitrary and illogical.	We constructed the five themes described using framework analysis. Lines 171 – 183 describe the steps involved in detail, highlighting how the analytical framework was developed based on our research questions, knowledge of theory and prior research, familiarisation with the data, and line by line coding.<br><br>We have used a reporting checklist (standards for reporting qualitative research (SRQR)) to guide our reporting of the qualitative analysis throughout to help ensure there is sufficient detail for the reader.
**(4) Results**<br>i. I suggest presenting the information of these 12 interviewees in the table and disclosing more critical information that can reflect their representativeness, such as professional background, which is very important for this study.	Unfortunately, we are not able to provide additional information about the 12 interviewees for confidentiality reasons (in keeping with our ethics application). We note this in the results section (lines 203 – 204) and provide general information on the background of the interviewees (e.g., geographical location, length of service, and remit; lines 204 – 208).
ii. The author should use Nvivo to analyze and present some primary quantitative data, such as crucial word frequency, which can make this study more complete. In the current manuscript, Nvivo is not necessary.	As this is a qualitative analysis, we do not think it is appropriate to include quantitative data such as word frequency. While quantitative data can be valuable in certain contexts, it does not align with the objectives of our study which prioritises qualitative methods to develop a nuanced understanding of commissioners' experiences of child and adolescent mental health, their perspectives on the needs of their populations, the challenges they face and their needs for support and data. Including quantitative data in qualitative studies like ours is actively discouraged by qualitative researchers (including ourselves) to ensure the focus remains on the richness and depth of participants' experiences.<br><br>NVivo is a data management tool and, as such, was essential for the current analysis – helping us to manage the qualitative data (i.e., line by line code data using the analytical framework developed).
**(5) Discussion**<br>The authors confine themselves to discussing the CAMHS system without whether it can be used in other countries. As mentioned in the title of this article, this is a study based on experience. However, the current manuscript has not yet achieved the author's purpose.	Thank you for your perspective on the discussion. In our paper, we use the term CAMHS to refer to specialist services for children and young people's mental health problems. Our paper focusses on exploring the perspectives and experiences of those commissioning these services, rather than on investigating the need or configuration of the services themselves, and we would respectfully consider that we have met this aim.

**2.Minor comments**<br>The references lack evidence from high-quality journals, significantly research articles.	We respectfully disagree with this comment. We include references to relevant reports and research articles published in high-quality journals (e.g., Journal of Child Psychology and Psychiatry, Lancet Psychiatry, European Child & Adolescent Psychiatry, BMJ Open).<br><br>If there is a relevant reference you believe we are missing, please let us know.
**Reviewer 2**	
My main comment has to do with the lack of "packaging" the study in a way that makes it understandable for an international audience. The framework of health systems research seems a bit absent. As a non-UK mental health professional, I ended up having many questions after reading, for example: How is the provision of care organized in the UK? I know of the NHS, but how does it work in practice? Who decides, and on what level, what is to be prioritized and what resources are allocated to the regional/local level? Policies are referred to, but it is unclear if these are just recommendations or if it they are expected to be followed. How is the concept of evidence base incorporated in policies? Are there political decisions somewhere along the way?<br><br>How is the responsibility for CYP mental health divided between the primary care level (GP) and the specialist level? Do schools have any responsibility for child health/child mental health? Are these commissioners involved only on the specialist care level, and if so – how do they communicate with other parts of the service provider system and decide who does what?<br><br>Please describe how an ICB is organized and what type of professionals usually are part of such boards and how they work. Even though it is justifiable not to describe the specifics of each informant in the paper, more contextual knowledge of the commissioner professional profile, role and mandate would help. It appears from lines 184-188 as if the commissioner role can be filled by various types of professionals – but who decides what competence is required? Any national guidelines/recommendations? Given that	Thank you. We have added additional contextual information to the introduction and methods section, describing how healthcare is organised in England and providing more detail on ICBs as requested.<br>In this section we also briefly describe the qualification and background of commissioners and provide information on provider collaboratives.<br>We hope this helps to provide the clarification needed.

variability across the country is described this made me curious.<br><br>A detail: I am not a native English speaker and had some difficulty to understand the term "remit" that is widely used. My dictionary did not help much but I interpret the use of the word as if it intends to mean mandate or range of responsibility– correct? What type of background are these professionals expected to have? What is the relationship between the individual commissioner and the care provider? How much responsibility has an individual commissioner and how much are they expected to just deliver "on order"? You describe a shift in roles towards more dialogue, but is the key responsibility to produce contracts? Without such a contextual understanding, it is hard to interpret the results.	
**<u>Objective and Method</u>**<br>An important part of qualitative research is to clarify the perspectives from which the study has been performed, i.e. the motivations for doing the study. Throughout reading the paper, I sometimes wonder if the purpose is to highlight the situation and complexity of a certain professional role ("professional wellbeing"), or to understand the process of commissioning services for CYP mental health is ("systems approach"). Both perspectives are justified but motives need to be clarified. It is stated that the objective is "to explore commissioners' experiences…" but still that can be done in relation to both aims outlined above.	We completely agree, it is important to be clear about the motivations for doing the study.<br><br>Our motivations are a mixture of the two you describe: we sought to better understand commissioners' experiences of commissioning child and adolescent mental health services ("systems approach", process of commissioning), their perspectives on the needs of their populations, the challenges they face ("professional wellbeing", situation and complexity of professional role) and their needs for support and data ("professional wellbeing", situation and complexity of professional role).<br><br>We have highlighted this at the end of the introduction (lines 106 – 109) along with our research questions (lines 110 – 113) which also map onto to these two concepts:<br><br>1. How do commissioners develop an understanding of the needs of their population? ("systems approach", process of commissioning)<br>2. How do commissioners plan and adapt services to meet population need? ("systems approach", process of commissioning)<br>3. What challenges do commissioners face in their roles, and how can they be better supported? ("professional wellbeing", situation and complexity of professional role)

With a self-selection procedure the limitation always is that the group of informants may not be representative, but that has been highlighted. Were all the informants selected and decided at the same time point, or did the authors consider adding more informants until saturation occurred? Did the authors consider triangulating the data collection and also do a survey, or to interview professionals with leadership responsibility in the provider chain?	We have added further detail to the methods describing our estimated sample size and method for obtaining this.<br><br>We recruited participants through two main methods: 1) advertisements through social media and commissioner networks; and 2) e-mails to ICBs within England (UK). Adverts and e-mails went out at roughly the same time, but responses from commissioners came back gradually between May – June 2023. We interviewed all those who expressed an interest in taking part within the study time frame.<br><br>We derived an estimated sample size of 12-16 participants using the concept of information power (Malterud, Siersma, & Guassora, 2016) as well as our own knowledge and experience of conducting similar, qualitative interviews. Within the study time frame we had reached this sample size target, being able to sample across commissioners working in different settings (e.g., urban and non-urban contexts whose experiences are likely to vary due to differences in available resources, funding, and need) and collecting data that was sufficient in addressing our research aims and questions.<br><br>We sought to understand commissioners' experiences of commissioning child and adolescent mental health services, their perspectives on the needs of their populations, the challenges they face and their needs for support and data. We felt qualitative methods were the most appropriate way to explore this given our research questions focused on understanding experiences and perspectives. However, it would be interesting for future research to consider expanding on this work to explore the provider point of view, as suggested.
Line 155-57: I did not understand sentence "Commissioners have provided input into the key messages developed from this study and are currently involved in reviewing commissioner and policy-facing outputs". Please clarify whether "commissioners" (informants or other commissioners) have influenced the interpretation of results of the study in any way (i.e. in this manuscript).	Thank you for requesting clarification. We agree this could be more clearly explained. Commissioners have not been involved in the interpretation of the findings in this manuscript, but are currently involved in the development of outputs for commissioner and policy audiences. We have amended the Patient and public involvement section to reflect this more accurately.
The study was financed by various grants, which are described by abbreviations (e.g. line 652 "TND's NIHR Advanced Fellowship" and "NIHR Academic Fellowship"). Please clarify what these refer to more clearly and also if the funding institute in some way had some	Thank you for requesting clarification. We have spelled out the abbreviations, and added a statement to clarify the role of the funder, who had no involvement in the design, conduct or interpretation of the findings.

interest in the outcome of the study, and/or if they were involved in the design, performance, or interpretation of results in any way.	
**Results**<br>Line 232: "…linked to the advent of provider collaboratives". It appears as if authors here refer to a common trend or was it just this commissioner who described a change in how s/he worked? Further down on the same page the term "traditional commissioning models" is mentioned. Should I understand this as if the traditional model was a pure procurement procedure and that this traditional model is being abandoned on a larger scale? (this is a good example when lack of insight into the UK model makes it harder to interpret the text).	Thank you for pointing out further clarification is needed. We agree this was lacking. We have added more information to the methods section to describe service provider collaboratives and how these differ from traditional commissioning models.
Line 283: The term "frequent major healthcare system reorganisations" is mentioned. Does the informant here refer to local reorganisations or is this something that changed all over the country at the same time?	Here we refer to reform in healthcare system across England. We have now referenced this reform in the methods section in 'study context' and made this clear in the sentence you referenced.
Line 477-478: "…many commissioners felt that prevention and early intervention were key priorities". Do they refer to their own activities here or do they point in other directions/at other organisations? This is also mentioned in a quote on line 520.	Thank you for pointing out this is unclear. Commissioners were referring to the need for greater focus on prevention and early intervention in child and adolescent mental health more broadly. We have added further detail to the manuscript to make this clearer:<br><br>Lines 520 – 521: "…many commissioners felt that prevention and early intervention were key priorities within child and adolescent mental health"
Line 479: Since the authors state that several informants referred to something called the Thrive model, it could facilitate for the reader is this model was briefly described here as well (and not only referred to).	We have added a short description of Thrive where it is mentioned (lines 523 – 525). We have also included a reference to the Thrive model for anyone who is interested to find out more.
**Discussion**<br>The discussion still is quite descriptive and repeats/summarises results more than analyses them and puts them into context. One merit of this paper is that it focuses an important part of the complex process of providing mental health services to CYP. However, with the lack of a clearer description of the analytical framework guiding the paper, it appears	Thank you for your comments. Please see our earlier responses in terms of the analytical framework of the paper. In the discussion we aim to represent and discuss our findings, which includes both the systems perspective and the professional role of the commissioner.

as if the interpretation of the findings mixes the systems with the professional role perspective.	
The second and third paragraph (540-555) discusses the various perspectives on the commissioner role – system oversight/leadership or contract management? Once this dual role is identified, as it is in the results part, it would be interesting to discuss the duality rather than just describing it again. For example: Is it even possible to exercise system leadership while at the same time "buying" services from it? If so, how should commissioners interact with the system and still protect system integrity? Are there any risks identified in relation to this duality? And how are patients' interests protected in the process?	Thank you for raising this point. We feel that whilst the discussion points suggested are interesting, they go beyond the scope of interpretation and discussion of this particular study. However, we do discuss the role that commissioners have in advocating for their populations, and have expanded our discussion of the use of community voice by commissioners and the implications of this – which also relates to the comment below.
Lines 557-580 discusses the process of identifying needs and commissioning services according to such needs. The interesting part here is the described variation in how the informants work, what data they have access to, how they value/not value certain types of data etc. Here a more system oriented discussion could be warranted. The observation (i.e. description of such existing variation) justifies a discussion if there is need for system development. Based on these findings, is there a need for a broader discussion on a standardized procedure/guidelines for commissioning services? I do not see the stakeholder perspective here, the focus is more on the challenges related to the professional role of being a commissioner. But what can stakeholders (i.e. children, youth and their families) expect in terms of how decision makers and commissioners arrange the way they work? Would it be relevant to recommend triangulation of data (epidemiological data, provider data, and dialogue with stakeholders as well as experts) before deciding on such important issues as service provision for mental health in youth?	Thank you for this suggestion. In our findings, commissioners do discuss the role of stakeholder perspectives, including the use of data from multiple sources, including professionals and providers, and engagement with communities. We have extended our discussion and implications section to suggest the need for greater transparency and whether there is a need for more guidance.
Lines 582-592: This paragraph also describes part of the health systems pointing at each other. This is a well known phenomena also in my country.	Thank you for this suggestion, and we agree this is an interesting discussion. We would suggest however that such discussion on organisation of services in outside the scope of this particular paper, which is

Here as well, it would be possible to discuss how the "system" could interact/develop in ways which ultimately would see to the whole care chain and how each part can contribute to whole. Are there any examples of alternative ways of working or organizing services that would make it easier for CYP to access services according to needs? I do not expect ultimate solutions anywhere, but maybe good (or better) examples can be identified? Or attempts to cross bridges between piecemeal solutions?	about representing the perspectives of commissioners and the commissioning role.

**** Response to Reviewer - Response to reviewers 190624.docx ****

VERSION 2 - REVIEW

**Reviewer**	**2**
**Name**	**Hellner, Clara**
**Affiliation**	**Karolinska Institute, Clinical neuroscience**
**Date**	**09-Aug-2024**
**COI**	**None**

Comments on MS bmjopen-2024-086403

Thank you for allowing me to read the revised version of this manuscript.

Many of the remarks from the first review have been addressed. However, both I and the other reviewer highlighted the need of explaining to an international audience why this study would be of interest for professionals outside of the UK system. I think the authors still fail to analyse their results from this perspective.

In the new version, there is more information on the professional background of commissioners, as well as an explanation of what an ICB is and how it works. There is also more info on provider collaboratives. The authors state that the perspective of the paper is both on the professional role and the system. However, I still find it focusing primarily on professional roles and less on the system, in particular the relationship between these two. It is still a study that mainly report what commissioners say about their professional role in the UK, which is fine given that the research objective was described in that way. But without an analysis of what these results actually mean in relation to the system in which these commissioners work, the results are of less interest. As a reader, one is left with many outstanding questions, such as if the commissioners' role needs to change in anyway and if so, how? Even though this is a study of the UK context, I still do not understand how a commissioner in the UK is expected to relate to guidelines, policies etc.

I also notice that the other reviewer asked for more references to systems outside the UK but the authors state that this is primarily a study on the UK context and thus not necessary to include information from other countries. If so, I would suggest publishing in a national journal.

There is also a problem with the conclusion in the abstract. The abstract now concludes that there is a need for "..greater investment in children and young people's mental health, an increased focus on early intervention and prevention..". These conclusions do not in a clear way relate to any of the research objectives since the questions are open and explorative but the conclusions are normative. In this conclusion, there are two questions hidden; (1) if more resources would impact the commissioner role, or (2) if more investment in early intervention and prevention would impact the commissioner role. None of these questions have been addressed in the study, and it sounds as if the authors here more join the general discussion of what is needed to support youth mental health in general. I do not doubt the finding that resources are scarce and vary in unjustified ways, but the focus of the paper was how commissioners experience their role. Commissioners of cancer care or geriatric care most probably also experience a lack of resources, or for that matter any commissioner with a responsibility for an area with great need. And what is "early intervention"? Early in age or early in disease? And what type of prevention? Prevention of abuse, school failure, poverty? The term is often used as a general call for action of something that would make life better for children and young people, but it remains to be proven what type of prevention would decrease the pressure on CAMHS and also how that would impact a commissioner.

In summary, I think the paper's focus on the commissioner role is very interesting and worth studying, but it lacks a stringent analysis of the findings. It is therefore of less interest for an international audience but would probably be of greater value for a UK audience.

VERSION 2 - AUTHOR RESPONSE

Response to reviewer

Thank you for taking the time to review our manuscript. Please see below for responses to each of the points raised.

Comments and suggested changes	Author response
Need to explain to an international audience why this study would be of interest for professionals outside of the UK system. Authors fail to analyse results from this perspective.	Thank you for your comments. In our paper, we draw out several areas where findings may be transferable. We have also added a sentence to the introduction to highlight this.

The authors state that the perspective of the paper is both on the professional role and the system. However, I still find it focusing primarily on professional roles and less on the system, in particular the relationship between these two. It is still a study that mainly report what commissioners say about their professional role in the UK, which is fine given that the research objective was described in that way. But without an analysis of what these results actually mean in relation to the system in which these commissioners work, the results are of less interest. As a reader, one is left with many outstanding questions, such as if the commissioners' role needs to change in anyway and if so, how? Even though this is a study of the UK context, I still do not understand how a commissioner in the UK is expected to relate to guidelines, policies etc.	Thank you for your thoughtful comments. We consider that our analysis reflects commissioners' perspectives on their roles within the system. We do feel unable however to extend the paper beyond its current scope in terms of recommendations, which would go beyond the data we collected.
More references needed to systems outside the UK but the authors state that this is primarily a study on the UK context and thus not necessary to include information from other countries. If so, I would suggest publishing in a national journal.	Thank you for your comments. We do make reference to international systems through the paper, and have added some additional references and discussion (e.g. on page 24). We do note that BMJ Open publishes many similar papers which report primarily on a single-country context, but would defer to the editor on whether this paper would be more suitable elsewhere.
The abstract now concludes that there is a need for "greater investment in children and young people's mental health, an increased focus on early intervention and prevention..". These conclusions do not in a clear way relate to any of the research objectives since	Thank you for raising this point. We agree, that the "need" is as reported by commissioners rather than a finding of the paper, and have amended the conclusion of the abstract.

the questions are open and explorative but the conclusions are normative.<br><br>In this conclusion, there are two questions hidden; (1) if more resources would impact the commissioner role, or (2) if more investment in early intervention and prevention would impact the commissioner role. None of these questions have been addressed in the study, and it sounds as if the authors here more join the general discussion of what is needed to support youth mental health in general. I do not doubt the finding that resources are scarce and vary in unjustified ways, but the focus of the paper was how commissioners experience their role. Commissioners of cancer care or geriatric care most probably also experience a lack of resources, or for that matter any commissioner with a responsibility for an area with great need.	 <br><br>Thank you. In this qualitative paper, we seek to represent the perspectives of commissioners, who report themselves that the issue of resources is one which impacts their work, and that they perceive that children and young people are seen as lower-priority in the system. We have checked through the Discussion and Conclusion and made some minor amendments to ensure this is clear. We agree that lack of resource is a common problem, and acknowledge this in the Discussion: "As with many public sector services, CAMHS commissioners also face challenges in delivering services in the face of constrained resources."
What is "early intervention"? Early in age or early in disease? And what type of prevention? Prevention of abuse, school failure, poverty? The term is often used as a general call for action of something that would make life better for children and young people, but it remains to be proven what type of prevention would decrease the pressure on CAMHS and also how that would impact a commissioner.	Thank you for your comments. We agree it is a very general term, which commissioners often used in a general sense. However, we do not make recommendations regarding early intervention or prevention in the paper.
The paper lacks a stringent analysis of the findings. It is therefore of less interest for an international audience but would probably be of greater value for a UK audience.	We respectfully disagree with your comment that the paper lacks a 'stringent analysis'. We used Framework Analysis to analyse data from our interviews and clearly describe this process in the methods (page 7). Framework analysis a systematic qualitative analysis method and the BMJ Open have published multiple papers that have employed this type of analysis.
